# Quick and Simple Evaluation of Sudomotor Function for Screening of Diabetic Neuropathy

**DOI:** 10.5402/2012/103714

**Published:** 2012-07-09

**Authors:** Chittaranjan S. Yajnik, Vaishali V. Kantikar, Amol J. Pande, Jean P. Deslypere

**Affiliations:** ^1^Diabetes Unit, King Edward Memorial Hospital Research Centre, Rasta Peth, Maharashtra 411011, Pune, India; ^2^Aesculape, Singapore

## Abstract

*Objective*. The aim of this study was to compare SUDOSCAN, a new device to evaluate sweat function (reflecting peripheral small C-fiber status), with conventional measures of peripheral and cardiac neuropathy in patients with type 2 diabetes. *Methods*. 265 diabetic patients were tested for symptoms and clinical signs of neuropathy using Michigan Neuropathy Screening Instrument (MNSI), vibration perception threshold (VPT) using biothesiometer, and cardiac autonomic neuropathy (CAN) using Ewing's protocol. Sudomotor function was investigated with SUDOSCAN through measurement of electrochemical skin conductance (ESC) of hands and feet. Lower ESC is suggestive of sudomotor dysfunction. *Results*. Lower ESC at feet was significantly associated both with increasing symptoms (MNSI A) and increasing score on physical abnormalities (MNSI B). Lower ESC at feet was also significantly associated with increasing VPT by biothesiometry (*P* < 0.01), and with higher number of abnormal CAN results (*P* < 0.05). ESC was associated with postural fall in blood pressure (sympathetic abnormality) (*P* < 0.05), but not with heart rate variability (HRV) tests (parasympathetic abnormalities). *Conclusions*. Sudomotor dysfunction testing may be a simple test to alert physicians to peripheral nerve and cardiac sympathetic dysfunction. Ease of performance could make it useful in a busy diabetic clinic. Further studies with hard clinical outcomes are indicated.

## 1. Introduction

Neuropathy is a common complication in diabetes mellitus (DM), with 60%–70% of patients affected over lifetime. Symptoms of neuropathy are very common, and subclinical neuropathy is more common than clinical neuropathy [1]. Neuropathy may remain undetected, and progress over time leading to serious complications. The most common clinical affection is peripheral neuropathy, affecting the feet. Autonomic nerve involvement is common but probably the most undiagnosed [2]. Clinical neuropathy is usually diagnosed by symptoms and by testing vibration sensation (tuning fork) and touch (Semmes-Weinstein Monofilament Examination, (SWME)) [3]. Tests for autonomic neuropathy are usually based on HRV but are difficult to perform and therefore rarely done in daily practice [4, 5]. It would be therefore useful to have an easy and quick screening test for peripheral and autonomic neuropathy.

Sweat glands are innervated by thin and unmyelinated sympathetic C fibers that could be affected early in the course of diabetes, as demonstrated in patients with prediabetes [6]. The consensus statement of the American Diabetes Association (ADA) includes sudomotor function in the diagnosis of early autonomic neuropathy in diabetes [7]. Several methods have been developed but the lack of easy, quick, and quantitative test to diagnose sweat dysfunction has restricted their widespread use in clinical practice [8–10]. SUDOSCAN is a non invasive device for easy, quick, and quantitative assessment of sudomotor function [11]. Combining direct current (DC) stimulation and reverse iontophoresis, it measures local conductance derived from electrochemical reaction between sweat chloride and nickel electrodes. It could be used to screen sympathetic system dysfunction which is common in subjects with impaired glucose tolerance (IGT) and diabetes [6, 8, 12–15].

We compared SUDOSCAN findings with conventional measures of peripheral and cardiac neuropathy in patients with type 2 diabetes, in a proof of principle study to investigate the usefulness of this measure in clinical practice.

## 2. Patients and Methods

### 2.1. Patients

The study was conducted in Diabetes Unit, King Edward Memorial (KEM) Hospital Research Centre, Pune, India from September 2009 to February 2010. KEM Hospital Research Centre ethics committee approved the study, and informed consent was obtained from each subject.

Type 2 diabetes patients between 21 and 75 yrs of age, with or without symptoms of neuropathy, were enrolled in the study. Exclusion criteria included treatment with drugs that would have an effect on the sympathetic system such as beta blockers (to avoid interference in CAN testing), amputation of arms or legs, implantation of electrical implantable devices (pacemaker/defibrillator), sensitivity to nickel or any other standard electrodes, history of seizures or epilepsy, presence of proliferative retinopathy, myocardial infarction (MI) and/or stroke in the past 6 months, arrhythmia's, treatment with anti-arrhythmic drugs, and any advanced systemic condition.

### 2.2. Methods

A medical officer examined the patients. Height, weight, waist, and hip circumference were measured using standardised methods and used to calculate body mass index (BMI) and waist hip ratio (WHR). Blood pressure was recorded in supine position after 5 minutes rest using oscillometry. Medication history (diabetes, cardiovascular, supplements, and other) was noted.

#### 2.2.1. Neuropathy Assessment

We used the MNSI questionnaire to record neuropathic symptoms (MNSI A) [16]. Clinical assessment included foot inspection (deformities, skin changes, and infection), ankle reflex testing, vibration sensation using 128 Hz tuning fork, and touch sensation perception using 10-g SWME (MNSI B). A composite score was calculated separately for symptoms (i.e., MNSI A) and for clinical examination (i.e., MNSI B).

VPT was measured on both sides (left and right) using a biothesiometer on the plantar side of the great toe on a continuous scale. We used mean of the two sides for analysis.

#### 2.2.2. Ewing's Cardiac Autonomic Function Tests

International Diabetes Federation (IDF) (2005) recommends the use of resting heart rate and heart rate response to provocation tests (lying-standing, Valsalva, deep breathing), and lying and standing blood pressure difference for diagnosis of CAN [17]. ADA has recommended only orthostatic hypotension and resting tachycardia for diagnosis of CAN [18]. We used orthostatic hypotension and HRV tests to diagnose CAN. Each test was carried out according to the standard procedure described by Ewing and Clarke [19] using a commercial electrocardiogram (ECG) system (CANS, Chennai, India).

- HRV during deep breathing (E/I Ratio): *R-R* intervals during inhalation and exhalation are calculated. The longest *R-R* interval is determined during expiration (*R-R* max) and the shortest interval during inspiration (*R-R* min). The ratio of the longest to shortest *R-R* is called as E/I ratio. Normal values ≥ 1.21.
- HRV to standing (30/15 Ratio): shortest *R-R* interval is measured after standing which is around the 15th beat. This is followed by bradycardia which is indicated by the longest *R-R* interval around 30th beat. The ratio of longest to shortest *R-R* is calculated which is called as 30/15 ratio. Normal values ≥ 1.03.
- HRV during Valsalva maneuver: in this test the ratio of longest *R-R* interval after maneuver to shortest *R-R* interval during the maneuver is calculated. Normal values ≥ 1.20.
- Blood pressure response to standing (orthostatic blood pressure response): the postural fall in the blood pressure is taken as the difference between the systolic blood pressure lying and standing. Normal fall is ≤20 mmHg.

#### 2.2.3. Measurement of Sweat Function

**Figure 1:**
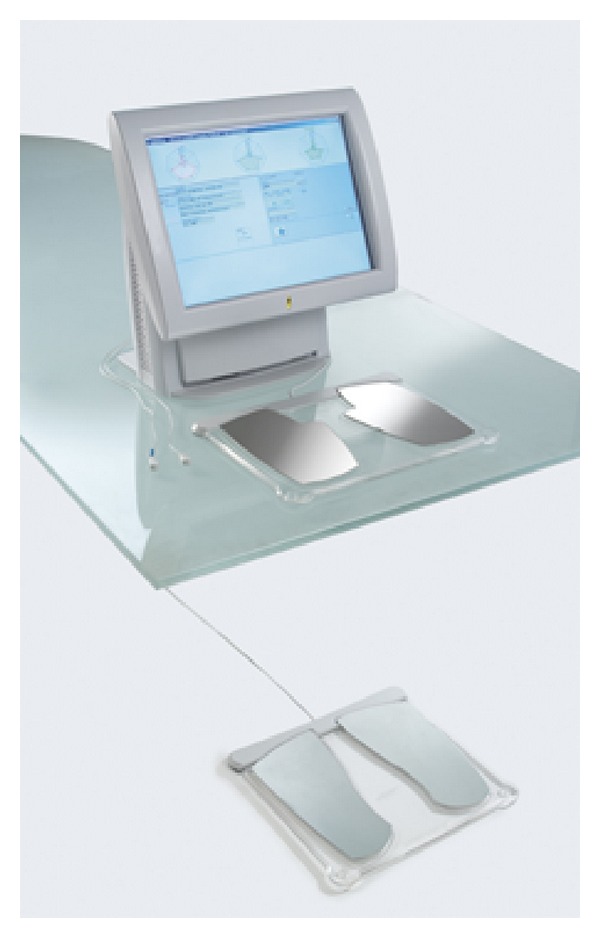
General presentation of the SUDOSCAN with the hands and feet electrodes and the master unit.

SUDOSCAN device (Figure 1) consists of two sets of electrodes for feet and hands which are connected to the computer for recording and data management. This is a non-invasive test and requires 2 minutes during which 4 combination of 15 different low voltages DC are applied. No subject preparation is required for this test. The subject places the palms of the hands and the soles of the feet on the electrodes. The machine measures ESC, that is the ratio of the current measured over the constant power applied expressed in microSiemens (*μ*S) for the hands, and the feet (right and left sides). We used mean of left and right readings for the analysis.

#### 2.2.4. Biochemical Analysis

Nonfasting blood sample was collected in EDTA vacutainer and processed to obtain plasma. Plasma aliquots were stored (−70°C) until further analysis. Hemoglobin was measured on whole blood on a Beckman Coulter Analyzer (AC.T diffTM, Miami, Fl, USA). Plasma glucose, uric acid, and creatinine, were measured on an automated biochemistry analyzer (Hitachi 902, Germany), using standard enzymatic methods. HbA_1c_ was measured using HPLC method (BioRad-D10, US) calibrated against (National Glycosylated standardization Program) NGSP. Urine albumin was measured using an immunoprecipitation assay, and albumin-creatinine ratio (UACR) was calculated.

#### 2.2.5. Statistical Analysis

Data are presented as mean (sd). Normality of the variables was checked, and appropriate transformations were done before analysis. following variables needed transformation: log transformation for VPT and HRV tests including 30/15 standing ratio and the Valsalva maneuver, log-log transformation for E/I Ratio, and square root transformation for MNSI A score. Agreement between left and right readings (VPT and ESC) was investigated using mean percent difference and coefficient of variation (CV). Simple linear regression analysis and Chi-square proportional trend test were used to study the association of ESC with continuous and categorical measurements of biological determinants (age, gender, duration of diabetes, etc.) and conventional measures of peripheral and cardiac neuropathy (such as MNSI, VPT, and CAN results). Agreement between MNSI, biothesiometer readings, and ESC values was studied using simple linear regression analysis. In this we express results as prediction interval by calculating percentage of observation outside the prediction limit. Low percentage indicates good agreement. Association between ESC and biological determinants was also tested using multiple linear regression analysis. Area under the curve (AUC) of the ROC curve was calculated to measure the efficiency of ESC in diagnosing patients with and without neuropathy against VPT [20]. Statistical analysis was performed using statistical package R 2.9.2.

## 3. Results

We studied 265 type 2 diabetic patients (149 males and 116 females) who were found eligible for the study and gave informed consent. Clinical and biochemical characteristics of the subjects are described in Table 1.

Thirty six patients (13%) showed microalbuminuria, 7 (2.6%) had macroalbuminuria. None had serum creatinine > 1.5 mg%, 53 (20%) had eGFR < 90 mL/min/1.73 m^2^, of whom 6 (2.2%) had eGFR < 60 mL/min/1.73 m^2^. Sixty patients (22.5%) had nonproliferative diabetic retinopathy (NPDR). Seventy-four patients had low levels of plasma vitamin B12 (<150 pmol/L) or were on vitamin B12 supplements, and they had similar nerve function (MNSI A, MNSI B, VPT and ESC) compared to those who had higher levels of vitamin B12 and are included in the analysis.

### 3.1. Peripheral Neuropathy

Two hundred and thirty (86.8%) patients had at least one symptom of neuropathy. One hundred and forty-nine patients (56.2%) had abnormal appearance on foot inspection, 57 (21.5%) had reduced ankle reflex, 41 (15.5%) showed reduced vibration sensation using tuning fork, and 15 (5.7%) showed reduced touch sensation using SWME on at least one foot. One hundred and forty-four patients (54.3%) showed MNSI B score >2 “Clinical neuropathy”. Patients with neuropathy were older, had longer duration of diabetes, and had higher VPT (biothesiometer) and lower ESC (SUDOSCAN) (Figure 3) (Table 2).

On biothesiometry, 90 (34.0%) patients had a VPT reading of ≤10 V which is considered normal, 93 (35.1%) patients had a reading between >10 and ≤15 V, 60 (22.6%) had a reading between >15 and ≤25 V, and 22 (8.3%) had a reading >25 V. Increasing VPT was associated with higher age and longer duration of diabetes (*P* < 0.001, both), higher HbA_1c_ concentration (*P* < 0.05), higher serum creatinine and lower eGFR, and with decreasing ESC (Figure 4) (*P* < 0.01, all).

### 3.2. Ewing's Tests for Cardiac Autonomic Neuropathy (CAN)

In 265 patients, at least three CAN test results were available: 52 (19%) had abnormal E/I ratio (<1.21), 84 (32%) had abnormal 30/15 ratio (<1.03), and 21 (8%) had orthostatic hypotension (>20 mmHg). Valsalva test was performed satisfactorily by only 171 patients, out of which only 1 was abnormal (<1.20). Of 171 on whom all test results were available, in 102 all tests were normal, 46 had one test abnormal, and 23 had two or more tests abnormal.

### 3.3. Sweat Function Measurements

**Figure 2:**
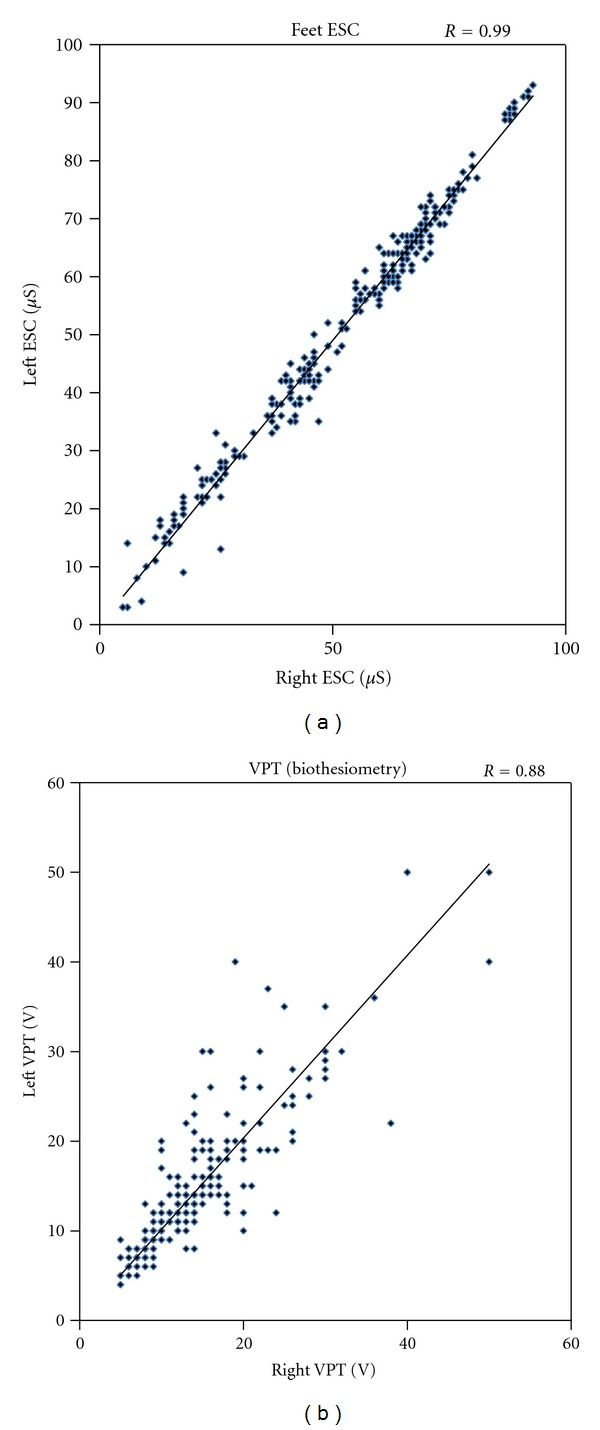
Agreement plot between left and right side measurements of ESC in feet using SUDOSCAN and vibration perception threshold (VPT) using biothesiometer. Figure shows a closer agreement in the ESC measurements compared to the VPT measurements.

**Figure 3:**
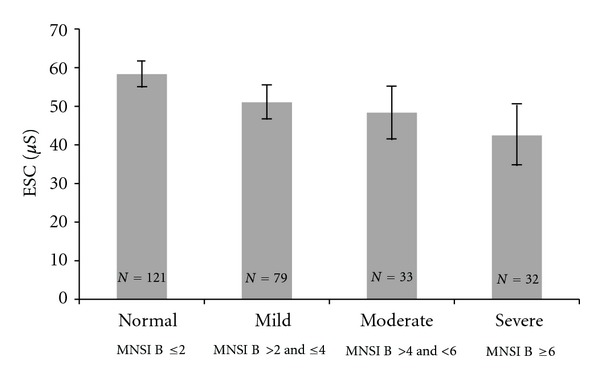
Figure shows association between Michigan Neuropathy Screening Instrument B (MNSI B) and Electrochemical Skin Conductance (ESC) in foot (SUDOSCAN). Increasing MNSI B was associated with decreasing ESC (*P* < 0.01). Each bar represents mean ± (SE *1.96).

**Figure 4:**
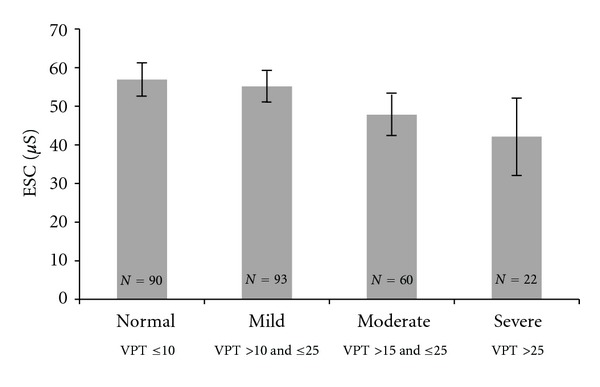
Figure shows association between vibration perception threshold (VPT) (biothesiometry) and electrochemical skin conductance (ESC) in foot (SUDOSCAN). Increasing VPT was associated with decreasing ESC (*P* < 0.01). Each bar represents mean ± (SE *1.96).

Conductance measurements were normally distributed in hands and feet. Mean percent difference between measurements on right and left side was 9.5% for hands and 6.0% for feet while it was 14.2% for VPT measurements (Figure 2). The CV for right and left side measurements was 6% for hands ESC, 4% for feet ESC, and 10% for VPT.

For further analysis, only the feet ESC results are used, and similar results were observed for hands ESC.

Table 3 shows clinical and biochemical characteristics, and results of conventional neuropathy testing by categories of decreasing feet ESC measurements. Lower ESC readings were significantly associated with higher age, longer duration of DM, higher HbA_1c_, and lower hemoglobin concentrations, but were not related to gender, BMI, WHR, non-fasting plasma glucose, uric acid, creatinine concentrations and eGFR. On multiple linear regression analysis, lower ESC was independently associated with higher age and higher HbA_1c_ (*P* < 0.05, both), but not with gender, anthropometric and other biochemical parameters.

Lower ESC was significantly associated with both increasing symptoms (MNSI A) (*r* = −0.12, *P* < 0.05), increasing score on physical abnormalities, suggestive of peripheral neuropathy (MNSI B) (*r* = −0.26, *P* < 0.01), and increasing score on VPT (*r* = −0.23, *P* < 0.01). Compared to patients with ESC ≥ 40 *μ*S, patients with ESC < 40 *μ*S were more than two times likely to have MNSI B score > 2, (OR = 2.3 (1.28–4.14)), more than three times likely to have VPT > 25 V (OR = 3.97 (1.61–9.99)), and more than 4 times likely to have 2 or more CAN tests abnormal (OR = 4.41 (1.72–11.29)). Of the four CAN tests, only postural fall in blood pressure was associated with lower ESC (*r* = −0.17, *P* < 0.05).

Agreement between MNSI, VPT, and feet ESC was tested by simple linear regression analysis with prediction interval. We regressed MNSI (A and B score) and VPT on ESC and calculated the predicted VPT and MNSI measurements for any observed value of ESC. Percentage of observations outside prediction interval was 2.6% for MNSI A, 1.5% for MNSI B, and 4.5% for VPT, which indicates good agreement.

Efficiency of sweat function measurement in diagnosing patients with and without neuropathy was checked using ROC curve. The AUC of ESC, calculated against continuous scale of VPT, was 71% at VPT = 21 V. The sensitivity and specificity, calculated on the continuous scale of ESC, were 73% and 62%, respectively, at ESC = 52 *μ*S which indicates that 73% of neuropathy patients and 62% of without neuropathy patients were correctly diagnosed by SUDOSCAN.

## 4. Discussion

In this proof of principle study in diabetic patients, we found that lower ESC on SUDOSCAN (reflecting sweat gland dysfunction) was significantly associated with symptoms of peripheral neuropathy and conventional clinical tests of vibration perception and monofilament touch. The association of ESC with vibration perception (biothesiometer) was continuous and graded, similar to the findings of a previous study performed in France [21]. Lower ESC was also associated with postural hypotension (reflecting sympathetic dysfunction) but not with HRV tests (reflecting parasympathetic dysfunction). Our results suggest that SUDOSCAN could be used as a simple noninvasive test for screening diabetic neuropathy and sympathetic CAN along with conventional tests. It is not a substitute for conventional neuropathy testing but would alert the physician to perform more careful testing for autonomic neuropathy.

SUDOSCAN evaluates sudomotor function by measuring ESC resulting from an electrochemical reaction between sweat chloride and nickel electrodes after a low DC stimulation. Importance of sweat chloride in this reaction was well demonstrated in a study performed with cystic fibrosis (CF) patients who had significantly higher conductance compared with healthy subjects [22]. Sweat gland function is controlled by sympathetic C fibers which might be affected early in the process of pathogenesis of diabetes. Sweat gland dysfunction has been demonstrated in patients with early diabetes using different methods including skin biopsies [8–10, 12–15]. Subjects with impaired glucose tolerance have lower ESC compared to those with normal glucose tolerance, and subjects with diabetes have even lower conductance [23]. Clinical tests of diabetic neuropathy are mostly based on testing of peripheral nerves, usually involving the large type A alpha and beta-myelinated nerve fibers (vibration and touch sense). However, the unmyelinated, thin type C fibers of sympathetic nervous system are not tested. SUDOSCAN offers a simple test to evaluate these fibers. Increasing age, duration of diabetes, and poor glycemic control (HbA_1c_) predict low ESC, indirectly supporting that it is a reliable measure of diabetic neuropathy.

Tuning fork and monofilament tests are easy to perform but qualitative. Biothesiometry is quantitative. But all these tests are subjective, requiring patient's attention and cooperation [24], and there is asymmetry of the results (Figure 2). On this background, the symmetry of ESC results on the two sides is remarkable. This could be explained by the “objective” nature of the investigation in a symmetrical involvement of peripheral nervous system. The recommended tests for autonomic neuropathy (Ewing's HRV tests) are rarely used because they are complex to perform, are time consuming, require a well-trained investigator, and involve considerable patient participation (American Diabetes Association, 2011). Newer methods like the use of a Holter machine and time and frequency domain analysis are not performed on everyday basis. On the other hand, SUDOSCAN measurement takes 3 minutes, does not require patient preparation or active participation, and can be performed by a nonmedical person in a busy clinic.

Admittedly, this proof of principle study has some limitations in applicability because of the design and methodology. The need to compare the results with conventional HRV tests (Ewing's protocol) necessitated exclusion of patients who were on medications which could affect HRV. It is also true that Ewing's protocol is somewhat outdated though it remains the “standard” method for autonomic testing. The use of a DC in the testing required exclusion of patients who suffered from seizures or were carrying an electrical medical device. Patients with foot ulcers or amputation had to be excluded because of the technical requirement of the test. Comparison of vibration perception (biothesiometer) and sudomotor function meant that we compared affection of large, myelinated type A fibers with that of small, unmyelinated type C fibres. However, this comparison allowed us to test the performance of a novel method with established clinical methods. It would be informative to compare SUDOSCAN with other methods of sweat gland function like QSART (reference method but difficult to perform on large scale) [8] or Neuropad (not quantitative) [9, 10], which we plan to do in future. We feel that SUDOSCAN measurements will expand the scope of future investigations to study tissue damage in early stages of diabetes by a relatively simple, quick and noninvasive test.

Further clinical application of sudomotor testing in a diabetic clinic will depend on conducting larger unbiased studies in representative patient populations and relating the findings to varied clinical outcomes including foot ulceration, amputations, cardiovascular events, and sudden death, in all of which autonomic neuropathy is thought to play a significant role.

## 5. Conclusion

Assessment of sudomotor function using SUDOSCAN, a quick and noninvasive test, could be considered as an additional screening test to alert physicians to peripheral nerve and cardiac sympathetic dysfunction. Ease of performance could make it useful in a busy diabetic clinic to identify patients with small fibre neuropathy who may be at higher risk of foot problems or cardiac neuropathy.

## Figures and Tables

**Table 1:** Basic clinical and biochemical characteristic of study subjects.

Clinical characteristics	
Gender	Male (149); female (116)
Age (yr)	53.08 (9.07)
Duration of diabetes (yr)	9.32 (7.09)
Weight (kg)	67.55 (10.59)
Height (cm)	1.59 (0.08)
BMI (kg/m^2^)	26.45 (3.70)
Waist-hip ratio (WHR)	0.95 (0.07)

Biochemical characteristics	

Haemoglobin (gm%)	12.49 (1.67)
Random blood glucose (mg%)	177.50 (76.01)
HbA_1c_ (%)	8.54 (1.82)
Uric acid (mg%)	4.67 (1.19)
Creatinine (mg%)	0.74 (0.18)
eGFR (mL/min/1.73 m^2^)	111.10 (28.62)
UACR (mcg/mg creatinine)	49.95 (238.76)

Values are mean (sd).

**Table 2:** Comparison of clinical, biochemical characteristics and ESC measurement in patients without (MNSI B score ≤2) and with (MNSI B score >2) clinical neuropathy.

	MNSI B score ≤2	MNSI B score >2	*P* value
	(*N* = 121)	(*N* = 144)	
Gender	Males (74), females (47)	Males (75), females (69)	
Age (yr)	49.82 (8.93)	55.81 (8.27)	<0.001
Duration of DM (yr)	6.85 (5.97)	11.40 (7.30)	<0.001
BMI (kg/m^2^)	25.93 (3.50)	26.88 (3.82)	0.03
Waist-hip Ratio	0.95 (0.07)	0.95 (0.08)	0.74
Hemoglobin (gm%)	12.82 (1.72)	12.20 (1.57)	<0.01
Random glucose (mg%)	177.07 (74.60)	177.92 (77.43)	0.92
HbA_1c_ (%)	8.34 (1.75)	8.70 (1.87)	0.11
Uric acid (mg%)	4.71 (1.24)	4.64 (1.16)	0.62
Creatinine (mg%)	0.74 (0.17)	0.74 (0.20)	0.98
eGFR (mL/min/1.73 m^2^)	113.22 (28.08)	109.38 (29.15)	0.27
VPT (volts)	10.40 (4.52)	17.11 (7.93)	<0.001
Hand ESC (*μ*S)	58.77 (19.84)	48.40 (20.90)	<0.001
Feet ESC (*μ*S)	58.32 (19.86)	48.49 (21.12)	<0.001

Values are mean (sd). *P*-value calculated using *t* test. ESC and VPT readings are mean of left and right sides.

**Table 3:** Association between ESC measurements in feet and clinical, biochemical and conventional neuropathic characteristics in type 2 diabetic patients.

	ESC > 60 (*μ*S)	ESC 40 ≥ & ≤ 60 (*μ*S)	ESC < 40 (*μ*S)	*P* value
	(*N* = 130)	(*N* = 67)	(*N* = 68)	
Gender	M (74), F (56)	M (38), F (29)	M (37), F (31)	
Age (yr)	51.0 (11.5)	53.0 (11.5)	57.0 (10.5)	<0.001
Duration of DM (yr)	6.0 (10.0)	8.0 (10.0)	12.5 (10.5)	<0.001
BMI (kg/m^2^)	25.9 (5.1)	26.7 (5.2)	26.3 (5.3)	0.88
Waist-hip ratio	0.95 (0.09)	0.96 (0.11)	0.96 (0.11)	0.43
Hemoglobin (gm%)	12.5 (2.3)	12.5 (1.9)	12.1 (2.3)	<0.01
Random glucose (mg%)	169.0 (89.7)	171.0 (99.5)	159.0 (106.7)	0.76
HbA_1c_ (%)	8.0 (2.0)	8.3 (2.6)	8.6 (2.7)	<0.01
Uric acid (mg%)	4.6 (1.7)	4.6 (1.6)	4.5 (1.5)	0.42
Creatinine (mg%)	0.7 (0.3)	0.7 (0.2)	0.8 (0.3)	0.47
eGFR (mL/min/1.73 m^2^)	111.0 (34.9)	110.0 (38.3)	97.2 (32.0)	0.10
MNSI B score >2 [*n *(%)]	59 (45%)	38 (56%)	47 (69%)	<0.01
VPT (Volts)	11.5 (7.0)	13.0 (6.5)	15.0 (12.2)	<0.001
Patients with all Ewing tests performed (*N* = 171)	87	52	32	
≥2 Abnormal Ewing test [*n* (%)]	9 (10%)	4 (8%)	10 (31%)	0.01

Values are median (IQR) or *N* (%). For continuous variables *P* values are calculated using simple linear regression analysis, and for categorical variables using chi square proportion trend test in three ESC categories.
